# The Health Effects of US Unemployment Insurance Policy: Does Income from Unemployment Benefits Prevent Cardiovascular Disease?

**DOI:** 10.1371/journal.pone.0101193

**Published:** 2014-07-15

**Authors:** Stefan Walter, Maria Glymour, Mauricio Avendano

**Affiliations:** 1 Department of Social and Behavioral Sciences, Harvard School of Public Health, Boston, Massachusetts, United States of America; 2 Department of Public Health, Erasmus University Medical Center, Rotterdam, The Netherlands; 3 Department of Epidemiology, Erasmus University Medical Center, Rotterdam, The Netherlands; 4 Department of Epidemiology & Biostatistics, University of California San Francisco, San Francisco, California, United States of America; 5 Department of Social and Behavioral Sciences, Harvard School of Public Health, Boston, Massachusetts, United States of America; 6 LSE Health and Social Care, London School of Economics and Political Science, London, United Kingdom; Hunter College, City University of New York (CUNY), CUNY School of Public Health, United States of America

## Abstract

**Objective:**

Previous studies suggest that unemployment predicts increased cardiovascular disease (CVD) risk, but whether unemployment insurance programs mitigate this risk has not been assessed. Exploiting US state variations in unemployment insurance benefit programs, we tested the hypothesis that more generous benefits reduce CVD risk.

**Methods:**

Cohort data came from 16,108 participants in the Health and Retirement Study (HRS) aged 50–65 at baseline interviewed from 1992 to 2010. Data on first and recurrent CVD diagnosis assessed through biennial interviews were linked to the generosity of unemployment benefit programmes in each state and year. Using state fixed-effect models, we assessed whether state changes in the generosity of unemployment benefits predicted CVD risk.

**Results:**

States with higher unemployment benefits had lower incidence of CVD, so that a 1% increase in benefits was associated with 18% lower odds of CVD (OR:0.82, 95%-CI:0.71–0.94). This association remained after introducing US census regional division fixed effects, but disappeared after introducing state fixed effects (OR:1.02, 95%-CI:0.79–1.31).This was consistent with the fact that unemployment was not associated with CVD risk in state-fixed effect models.

**Conclusion:**

Although states with more generous unemployment benefits had lower CVD incidence, this appeared to be due to confounding by state-level characteristics. Possible explanations are the lack of short-term effects of unemployment on CVD risk. Future studies should assess whether benefits at earlier stages of the life-course influence long-term risk of CVD.

## Introduction

The US unemployment rate was 8.2% in early 2012, reaching 12.8 million people in January[1]. Unemployment can have several negative consequences including loss of income and pension benefits, increased tobacco and alcohol consumption[2]–[3], and changes in physical and mental health[4]–[7]. In particular, job loss during the years before retirement can critically disrupt savings and wealth accumulation[8], and is associated with increased risk of cardiovascular disease (CVD)[9]–[10]. An important, yet unexplored question, is whether US unemployment policies might reduce CVD risk by providing a safety net during unemployment spells.

In the United States, the Social Security Act of 1935 created the Federal State Unemployment Compensation Program, providing temporary wage replacement for workers who experience involuntary job loss[11]. Unemployment income is a major US welfare policy and often provides the primary source of income for recently unemployed individuals. Several studies have examined the association between income and CVD[12]–[17], but no studies have examined whether unemployment income is associated with reduced CVD risk.

The Federal-State Unemployment Insurance Program provides unemployment benefits to eligible workers who are unemployed through no fault of their own (as determined under State law), and meet other eligibility requirements of State law. It is a complex programme and states have flexibility along several dimensions: Each State administers a separate unemployment insurance program within guidelines established by Federal law. Eligibility for unemployment insurance, benefit amount and duration are determined by the State law under which unemployment insurance claims are established. To be eligible, workers must meet the State requirements for wages earned or time worked during an established period referred to as ‘base period’, which in most states is the first four out of the last five completed calendar quarters prior to the time that the claim is filed. Benefits are generally based on a percentage of an individual's earnings over a recent 52-week period up to a state maximum amount. In most states, benefits can be paid for a maximum of 26 weeks, and they are subject to Federal income taxes[18]. Unemployed workers are most likely to benefit from the unemployment program. However, the health benefits of unemployment income may extend to others, by providing all workers and their families with a sense of financial security. Given the links between psychological distress and CVD[19]–[24], if reductions in financial worries alleviate depression or anxiety, unemployment benefits may thereby lead to lower CVD risk among the general population regardless of employment status.

Identifying the causal effect of unemployment income is challenging because of strong selection into unemployment: if less healthy individuals are more likely to lose their job and claim unemployment benefits, the association between receiving unemployment income and CVD incidence would underestimate the true effect of unemployment benefits on CVD risk. An innovative approach to address this bias is to exploit the large variation in unemployment policies across US states. Each state has autonomy to define unemployment program eligibility and maximum compensation benefits. As a result, maximum duration and unemployment benefits vary considerably across states over the last decades. These variations over time are independent of an individual's social standing or previous health and offer a unique opportunity to examine the impact of unemployment benefits on CVD risk.

In this paper, we aim to isolate the impact of a single feature of the unemployment benefit programme on CVD incidence: the generosity of maximum unemployment benefits a worker is entitled to after job loss. We focus on this feature because it can be easily operationalized and compared across states, and because it reflects the comprehensiveness of the programme as a whole. Other components of the programme are important but they are difficult to operationalize in a comparable way across states. While the impact of the programme will also depend on benefit uptake, the latter is determined by individual characteristics that may be correlated with health. In contrast, changes in state benefit generosity are in principle uncorrelated with individual characteristics, which provides a potential natural experiment to assess the impact of benefit generosity on health.

To achieve this aim, we linked individual-level data from the Health and Retirement Survey (HRS) to yearly state-level data on maximum unemployment benefit laws to test the hypotheses that more generous unemployment benefits are associated with lower incidence of first and recurrent CVD in the US population as well as among unemployed workers. To control for differences across states, we estimated the effect of changes in the generosity of unemployment benefits on CVD risk using state-fixed effect models.

## Methods

The HRS study was approved by the University of Michigan human subjects committee. The current report describes secondary analyses of de-identified data and is therefore exempt from human subjects review. State of residence data, used in the current analysis, is considered a restricted data element, and special data security protocols are in place for these data; these security protocols were approved by the Harvard School of Public Health human subjects committee.

### Sample

The HRS is a longitudinal survey of US adults aged 50 and older and their spouses. Additional study details are available elsewhere[25]. The HRS sample was selected using a multi-stage area probability sample design of the US population, with enrolment staggered by birth cohort. We used cohort members enrolled in 1992 (Original HRS cohort, age-eligible born 1931–1941), 1998 (“War Babies” born from 1942–47), and 2004 (“Early Baby Boomers” born from 1948–1953). Response rates were high and ranged from 70% for the 1942 to 1947 birth cohort enrolled in 1998, to a high of 82% for the 1931 to 1941 birth cohort enrolled in 1992, without major differences by demographic factors. The majority of baseline interviews were face-to-face. Biennial interviews (or proxy interviews for decedent participants) were conducted through 2010, with wave-to-wave retention rates of around 90%. The study sample included all HRS participants aged 50–65 at some point between 1992 and 2010. From a total of 17,169 eligible participants, 182 were excluded because of missing information on state of residence or because they lived in one of five states with less than 50 individuals in the sample (Alaska, Hawaii, Rhode Island, South Dakota and Vermont). We excluded 49 participants with missing information on CVD, 753 participants with missing information on state of residence prior to the assessment of CVD, and 77 participants with missing information on other covariates. The total sample analyzed included 16,108 participants.

### Cardiovascular disease

CVD was defined as any stroke or heart disease (incident or recurring) based on self-reports of a physician's diagnosis in the two-year period preceding interview. New enrollees were asked whether a doctor had ever told them that they had had a heart attack, coronary heart disease, angina, congestive heart failure, or other heart problems. Participants were separately asked the same question regarding stroke. To assess incidence of new events, participants –or their proxies for deceased members, typically spouses -were asked every two years whether they had had a new diagnosis since last interview.

### Unemployment Insurance Policy: Maximum unemployment benefits

Income benefits received during an unemployment spell are correlated with factors potentially associated with health such as employment histories, previous earnings, and earlier unemployment spells. Therefore, the association between individual benefit receipt and CVD does not reflect the causal impact of unemployment benefits but is confounded by selection into unemployment and benefit claiming. Instead of using individual-level unemployment income received during unemployment spells, we collected data on the maximum unemployment benefits residents would be entitled to receive during unemployment spells according to the unemployment laws in their state of residence. The rationale for this approach is that maximum benefits influence the amount of benefits individuals will ultimately receive during an unemployment spell, but they are not influenced by individual's health as they are the result of state policy changes. Although this approach does not enable us to assess the direct impact of receiving benefits on CVD, it enables us to assess the impact of changes in unemployment benefit policies on CVD risk.

Yearly data on the duration and maximum amount of unemployment benefits individuals are entitled to receive were obtained from the US Department of Labor (http://www.oui.doleta.gov/unemploy/statelaws.asp, accessed July 2010). The maximum unemployment benefit was defined as the maximum monthly benefit (in dollars) multiplied by the maximum number of months a worker who becomes unemployed through no fault of their own would be entitled to receive. The amount is specific for each state and year. The actual unemployment income received by an individual who becomes unemployed depends on his or her salary while employed, the duration of prior employment, and the duration of unemployment. In order to avoid selection bias due to these and other variables, we used unemployment benefit entitlements at the state level, rather than the actual benefits received by each individual.

To account for price changes, all amounts were adjusted to 2006 US dollars using the consumer price index (CPI). In order to account for non-linear effects of maximum benefits, we used the natural logarithm of maximum benefits (log(maximum benefits/1000 USD_2006_)) as the main independent variable.

### Covariates

All models controlled for age, gender, respondent's years of education, mother's educational attainment (>8 years, < = 8years), father's educational attainment (>8 years, < = 8years), race (white, African American, other), Hispanic ethnicity, and time-varying marital status (married, separated/divorced, widowed, and never married). Missing values for marital status were imputed by carrying forward the last known value, which was typically not further than two years. In addition, we included an indicator variable for year of CVD assessment to account for secular trends in CVD incidence rates. Previous evidence suggested important health differences between HRS participants who were able to report on the education of their parents and those who did not know their parents' education, probably reflecting whether the respondent lived with both of his/her parents in childhood. For parental education, we therefore created a missing category (“unknown parents' education”). Employment status was assessed each wave by asking participants whether they were employed, unemployed, retired, disabled, or out of the labor force. Lagged employment status was used in all models to assure that it preceded the onset of cardiovascular disease and fell in the same time spell as the maximum unemployment benefits.

### Estimation Methods

We used Generalized Estimation Equations (GEE) to model CVD as a function of employment status, state maximum unemployment benefits and confounders. Models used a logit link and an unstructured working correlation matrix to account for repeated measures across waves. Because the HRS study is nationally representative the corresponding sampling weights were applied. In order to account for the possible time lag necessary for income benefits to influence CVD risk, we related CVD diagnoses at each wave to unemployment benefits in the preceding wave, approximately two years earlier. The analysis was conducted in five steps. We first modeled CVD as a function of lagged state maximum unemployment income benefits adjusting for confounders but without state fixed effects. This model exploits variation in unemployment benefits across states. In the next set of models, we adjusted for US census regional division (Midwest, Northeast, South, and West). In the third model, we included state fixed effects to control for all time-invariant differences across states. The major advantage of fixed effects methods[26] is that by differencing out variability within states, it is possible to control for all time-invariant differences across states (characteristics that vary across states but not over time). In the final model, we included information on within state changes in percentage of high school graduates, real average income, and the unemployment rate for person between 30–64 years of age in addition to state-level fixed effects.

To assess whether benefits mitigate the impact of unemployment on CVD, we implemented models that incorporated an interaction between state-specific maximum unemployment benefit and employment status. In sensitivity analyses, we examined alternative lag periods between the unemployment benefits and CVD rates.

## Results

Out of 17,169 eligible participants, 16,108 had complete data for at least one wave (Table 1). Of 12,482 CVD events reported between 1994 and 2010, 4,218 were first diagnoses. Participants had a median age of 55 (inter quartile range (IQR): 52–57 years) when first interviewed. Over half (55%) were female, and the median years of schooling were 12 (IQR: 12–14.5).

**Table 1:** Descriptive statistics at baseline, Health and Retirement Study, ages 50 to 65.

	Mean/n	SD/%
N	16108	
Maximum unemployment benefit ×1000 (2006 USD)	9.28	(0.02)
Age in Years	54.9 (0.02)	(0.02)
Gender (female)	8845	(54.9%)
Years of Education	12.46	(0.02)
Race		
White	12568	(78.2%)
African American	2688	(16.7%)
Other	852	(5.3%)
Ethnicity		
Non-Hispanic	14524	(90.2%)
Hispanic	1584	(9.8%)
Mother's Education		
Missing	1542	(9.6%)
>8years	5780	(35.9%)
< = 8years	8786	(54.5%)
Father's Education		
Missing	2362	(14.6%)
>8years	6323	(39.3%)
< = 8years	7423	(46.1%)
Marital Status		
Missing	740	(4.6%)
Married	11270	(70.0%)
Never Married	664	(4.1%)
Widowed	862	(5.4%)
Separated/Divorced	2571	(16.0%)
Work Status		
Missing	687	(4.3%)
Employed	10532	(65.4%)
Unemployed	408	(2.5%)
Retired	2281	(14.2%)
Disabled	697	(4.3%)
Not in Labor Force	1503	(9.3%)

**Figure 1:**
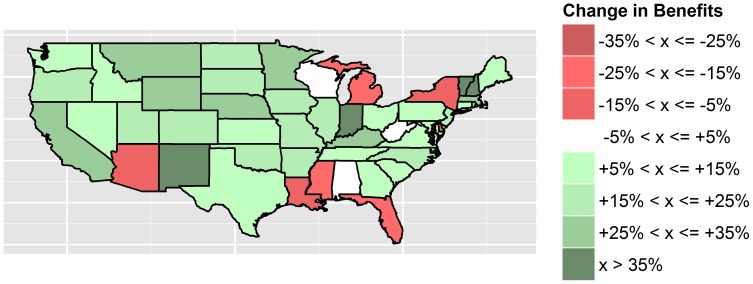
Percentage change in maximum unemployment compensation benefits (adjusted for differences in prices) in US states between 1992 and 2008.

The median maximum income benefit (in 2006 US dollars) an individual was entitled to receive if unemployed was US$8,840 (IQR: US$7,770–US$10,460). However, there were large differences across states. For example, in 1992, there was a three-fold difference in the benefit level between Alabama (US$3,900) and Massachusetts (US$13,320). Large variations were also evident in the evolution of benefits over time. Figure 1 shows the percentage change in maximum unemployment benefits in each US state between 1992 and 2008. During this period, many states reduced maximum unemployment benefits, while some states increased benefits. Only a few states maintained constant unemployment benefit levels. To illustrate (in 2006 USD), Arizona reduced unemployment benefits from US$ 6,537 in 1992 to US$ 5.843 in 2008, while Massachusetts increased benefits from US$19.140 in 1992 to US$25,282 in 2008.

Table 2 summarizes results of models examining the impact of unemployment benefits on CVD incidence. Female gender, younger age, higher educational level and Hispanic ethnicity were associated with reduced odds of CVD events. In models that did not include state-fixed effects (model 1), an 1% increase in the two-year lagged value of maximum unemployment benefits was associated with reduced odds of CVD (Odds Ratio (OR) = 0.82, 95%-Confidence Interval (CI): 0.71–0.94). This association was virtually unchanged in models that incorporated US census region divisions. However, incorporating state fixed effects, the association between state unemployment benefits and CVD risk disappeared (model 3, OR = 1.0.2, 0.79–1.31). Adding time-changing state level variables, namely percentage of high school graduates, real average income, and unemployment among the working age population, had very little impact on the estimated effect of benefits on CVD incidence (model 4, OR = 1.04, 0.77, 1.39). In sensitivity analyses, we assessed whether results were sensitive to the lag period used to define exposure to unemployment benefit laws. Estimates based on contemporaneous as well as four- and six-year lagged levels of unemployment benefits were very similar to original estimates based on a two-year lag (results not shown), suggesting that changes in maximum unemployment benefits were not associated with CVD risk. We furthermore tested different correlation structures (autoregressive, exchangeable) and found very similar results to those based on the unstructured correlation matrix used in our analyses (results not shown).

**Table 2:** Maximum unemployment benefit levels and CVD incidence, Health and Retirement Survey, ages 50 to 65.

	Model 1		Model 2		Model 3		Model 4	
	OR	(95% CI)	OR	(95% CI)	OR	(95% CI)	OR	(95% CI)
**Maximum unemployment benefit**								
log[maximum benefits/1000 USD2006]	0.82	(0.71, 0.94)	0.85	(0.72, 1.00)	1.02	(0.79, 1.31)	1.04	(0.77, 1.39)
**Age in Years**	1.05	(1.05, 1.06)	1.05	(1.05, 1.06)	1.05	(1.05, 1.06)	1.04	(1.04, 1.05)
**Gender** (female vs male)	0.72	(0.66, 0.78)	0.72	(0.66, 0.78)	0.72	(0.66, 0.78)	0.73	(0.67, 0.80)
**Years of Education**	0.93	(0.92, 0.95)	0.93	(0.92, 0.95)	0.94	(0.92, 0.95)	0.93	(0.92, 0.94)
**Race**								
White	1	Ref.	1	Ref.	1	Ref.	1	Ref.
African American	1.07	(0.95, 1.20)	1.05	(0.94, 1.18)	1.06	(0.95, 1.20)	1.14	(1.01, 1.30)
Other	1.09	(0.87, 1.34)	1.11	(0.90, 1.37)	1.11	(0.90, 1.38)	1.20	(0.95, 1.51)
**Ethnicity**								
Non-Hispancic	1	Ref.	1	Ref.	1	Ref.	1	Ref.
Hispanic	0.60	(0.51, 0.71)	0.62	(0.52, 0.74)	0.65	(0.54, 0.78)	0.66	(0.51, 0.81)
**Mother**'**s Education**								
Missing	1	Ref.	1	Ref.	1	Ref.	1	Ref.
>8years	1.04	(0.87, 1.23)	1.04	(0.87, 1.24)	1.04	(0.87, 1.24)	1.04	(0.86, 1.25)
< = 8years	1.08	(0.91, 1.29)	1.08	(0.91, 1.28)	1.08	(0.91, 1.29)	1.08	(0.89, 1.29)
**Father**'**s Education**								
Missing	1	Ref.	1	Ref.	1	Ref.	1	Ref.
>8years	0.89	(0.76, 1.03)	0.90	(0.77, 1.04)	0.90	(0.77, 1.05)	0.88	(0.74, 1.03)
< = 8years	1.03	(0.89, 1.19)	1.03	(0.89, 1.19)	1.03	(0.89, 1.190)	1.00	(0.86, 1.17)
**Marital Status**								
Married	1	Ref.	1	Ref.	1	Ref.	1	Ref.
Never Married	1.02	(0.83, 1.24)	1.02	(0.84, 1.25)	1.01	(0.83, 1.24)	1.09	(0.88, 1.36)
Widowed	1.15	(1.04, 1.27)	1.15	(1.04, 1.28)	1.15	(1.04, 1.27)	1.15	(1.02, 1.29)
Separated/Divoreced	1.24	(1.12, 1.37)	1.24	(1.12, 1.38)	1.25	(1.13, 1.38)	1.31	(1.17, 1.46)
**Employment Status**								
Employed	1	Ref.	1	Ref.	1	Ref.	1	Ref.
unemployed	1.19	(0.92, 1.55)	1.19	(0.92, 1.55)	1.20	(0.92, 1.56)	1.24	(0.92, 1.68)
Retired	1.45	(1.35, 1.56)	1.45	(1.35, 1.56)	1.45	(1.35, 1.56)	1.52	(1.40, 1.65)
Disabled	2.10	(1.83, 2.41)	2.10	(1.82, 2.41)	2.10	(1.83, 2.41)	2.38	(2.06, 2.75)
Not in Labor Force	1.18	(1.05,1.32)	1.18	(1.05,1.32)	1.17	(1.04,1.31)	1.19	(1.05,1.35)

All models control for age, gender, educational attainment, race, ethnicity, parental educational attainment and marital status and employment status at the previous wave; model 2 additional includes census regional division fixed effects (Midwest, Northeast, South, West), model 3 incorporates state fixed effects, and model 4 additionally includes state-level GDP, proportion of high school graduates, and unemployment rate between age 30–64.

Table 3 shows results of models that incorporate an interaction between individual employment status and state benefit levels at each wave. Over the study period, 1003 participants experienced at least one unemployment spell. Unemployment was unrelated to the risk of first or recurrent CVD, and there was no significant interaction between employment status and changes in state income benefits (p = 0.35). In sensitivity analyses, we also examined interactions with educational level, but found no evidence of an effect of income benefits across individuals with different levels of education (p = 0.79) (Table 3, model 3).

**Table 3:** Interaction of maximum unemployment benefit levels with employment status and educational level, Health and Retirement Survey, ages 50 to 65.

	Model 1			Model 2			Model 3			Model 4		
	OR	(95% CI)	p	OR	(95% CI)	p	OR	(95% CI)	p	OR	(95% CI)	p
**Maximum unemployment benefit**												
Log(1000–2006USD)	0.85	(0.69, 1.06)	0.15	0.88	(0.70, 1.11)	0.30	1.05	(0.78, 1.43)	0.74	1.18	(0.84, 1.68)	0.35
**Employment Status**												
employed	1	Ref.		1	Ref.		1	Ref.		1	Ref.	
unemployed	1.72	(0.20, 14.42)		1.70	(0.21, 14.08)		1.71	(0.21,14.12)		3.21	(0.28, 36.83)	
retired	1.47	(0.87, 2.50)	0.05	1.47	(0.87, 2.49)	0.05	1.44	(0.85, 2.45)	0.04	2.04	(1.14, 3.67)	0.01
disabled	5.12	(1.61, 16.34)		5.16	(1.60, 16.59)		5.32	(1.65, 17.10)		8.03	(2.37, 27.14)	
not in labor force	1.74	(0.61, 4.95)		1.76	(0.62, 5.00)		1.74	(0.61, 5.01)		2.10	(0.62, 7.13)	
**Interaction**												
Benefit x employed	1	Ref.		1	Ref.		1	Ref.		1	Ref.	
Benefit x unemployed	0.85	(0.32, 2.25)		0.85	(0.32, 2.25)		0.85	(0.32, 2.24)		0.65	(0.22, 1.97)	
Benefit x retired	0.99	(0.78, 1.26)	0.40	0.99	(0.79, 1.26)	0.39	1.00	(0.79, 1.27)	0.35	0.88	(0.67, 1.14)	0.27
Benefit x disabled	0.67	(0.39, 1.14)		0.66	(0.39, 1.13)		0.66	(0.38, 1.12)		0.58	(0.33, 1.01)	
Benefit x not in labor force	0.84	(0.53, 1.34)		0.83	(0.52, 1.33)		0.83	(0.52, 1.34)		0.77	(0.45, 1.34)	
**Maximum unemployment benefit**												
Log(1000–2006USD)	0.88	(0.50, 1.53)	0.65	0.90	(0.51, 1.58)	0.71	1.10	(0.60, 1.99)	0.77	0.95	(0.49, 1.85)	0.88
**Education**												
Years of Education	0.94	(0.86, 1.04)	0.24	0.94	(0.86, 1.04)	0.24	0.95	(0.86, 1.04)	0.28	0.92	(0.82, 1.02)	0.11
**Interaction**												
Benefit x Years of Education	0.99	(0.95, 1.04)	0.80	1.00	(0.95, 1.04)	0.84	0.99	(0.95, 1.04)	0.79	1.01	(0.96, 1.06)	0.78

All models control for age, gender, educational attainment, race, ethnicity, parental educational attainment, and marital status; model 2 additionally controls for census regional division (Midwest, Northeast, South, West), while model 3 incorporates state fixed effects, and model 4 additionally includes state-level GDP, proportion of high school graduates, and unemployment rate between age 30–64.

## Discussion

We hypothesized that increased unemployment compensation benefits would confer health benefits for employed workers and their families by providing a sense of security, and it would reduce the impact of unemployment on CVD risk. Although states with more benefits had lower incidence of CVD, incorporating state-fixed effects, we find no significant association between changes in state unemployment benefits and CVD risk.

A possible explanation for this finding is that our study focuses on a sample of individuals 50 years and older. Although late-life unemployment is associated with poorer health[10], the effects of unemployment, and the potentially ameliorating impact of unemployment benefits, may be weaker in mature workers who have already established careers and accumulated financial resources[27]–[28]. As a consequence, older individuals may be less dependent on unemployment benefit provisions, as opposed to younger workers who have accumulated less wealth and may rely more on unemployment benefit provisions. On the other hand, recent evidence suggests that Americans in their 50's who became unemployed during the recent recession lost more of their monthly per-capita earnings than any other age group [29]. Furthermore, older workers are among the highest beneficiaries of unemployment benefits. During the recent recession, 59% of long-term unemployed workers aged 50–51 and 46% of those aged 62+ were receiving unemployment benefits, compared to 53% of workers 35–49 and 41% of workers 25–34. This suggests that unemployment benefits are disproportionately claimed by unemployed older workers. Nevertheless future studies should examine whether unemployment benefits may confer health benefits for younger workers.

In addition, unemployment benefits in the US are characterized by relatively low recipiency rates. Low recipiency might explain the lack of a statistically significant effect in our study. In 2005, for example, only 51% of job losers applied for UI benefits, but rates increase sharply with age: Among men aged 16–24, only 29% of job losers claimed UI benefits in 2005, compared to 60% of job losers aged 45 or older[30]. Low recipiency is systematically linked to variables that reflect UI statutes and administrative operations as well as differences in features of state labor markets such as unionization. This results in large variations in WBTU (weekly UI beneficiaries as a proportion of weekly unemployment) across states. For instance, long run averages of WBTU for the years 1967 to 1998 ranged from 0.16 in Florida and Virginia to .56 in Alaska[31].

Over the last decades, several studies have suggested that unemployment is associated with a variety of health outcomes including mortality, suicide, myocardial infarction, stroke, disability and long-term illness[4], [10], [32]–[38]. Several mechanisms have been proposed to explain these associations. Unemployment is associated with a substantial loss in earnings[39], but it may also influence health via several non-financial pathways such as chronic stress, reduced social interaction, decreased self-esteem and social recognition[4], and increased prevalence of smoking, drinking and physical inactivity[40]. The fact that we find no evidence that changes in state unemployment benefit policies influence CVD risk suggest that, at ages 50 and above, unemployment may influence CVD risk through some of these non-financial mechanisms, so that other policies than unemployment benefits may be more important in preventing CVD incidence. On the other hand, our results are at odds with previous evidence that among US elderly, an increase in the Supplemental Security Income (SSI) cash transfer program benefits is associated with a fall of 0.46 percentage points in the rate of disability among US adults aged 65 and older.[41] A possible explanation for this discrepancy is that CVD risk is less sensitive to temporary income support benefits than other outcomes such as physical disability and mental health. Our models examined relatively short-term effects of state changes in unemployment benefits. The development of heart disease and stroke is a function of cumulative risk exposure throughout the life-course, and may involve long etiologic periods. Lower unemployment benefits during the critical years of work below age 50 may thus be more important in the development of cardiovascular risk later on in life, while benefit levels at older ages may be less important. Future studies should assess whether life-time cumulative exposure to different levels of unemployment compensation benefits throughout earlier career years may have cumulative effects on CVD risk, which only manifest many years or decades later.

### Limitations of our study

As one of the first study to evaluate the impact of unemployment benefit policies on health in the US, conclusions from our study should be interpreted with caution. We do not have medical verification of CVD events. This may have introduced bias if CVD reporting varied in tandem with state unemployment benefit policies. However, we have previously shown that CVD incidence as measured in HRS compares well with incidence estimates from clinically verified studies[42]. Assignment of unemployment benefits for each individual was based on maximum unemployment benefit eligibility in the state of residence for a given year. We therefore did not estimate the direct effect of receiving benefits among the unemployed. Instead, our study assessed the impact of changes in unemployment maximum benefit policies on CVD incidence. This approach has the advantage of overcoming bias due to selection into unemployment, as well as illustrating the potential impact of changes in policy. However, because only a small fraction of the sample actually became unemployed and was eligible for unemployment benefits, our estimates may mask larger effects of benefits among those actually receiving unemployment benefits.

Another limitation is that this study exploits year to year variations in the generosity of unemployment benefits among older Americans only. It studies the estimates the short-term effects of unemployment and unemployment benefit generosity on cardiovascular disease, rather than the long-term effects where other have identified significant effects of involuntary job loss on myocardial infarction and stroke in this study sample[9]–[10]. Our findings cannot be translated to long-term effects or to a younger age group and both merit additional scientific investigation.

A main strength of our approach is the introduction of state-fixed effects, to control for time-invariant differences across states. The drawback of this approach is that we are only able to exploit within-state variations in unemployment benefit level over time. Because differences across states are much larger than differences over time within states, our identification strategy relies on medium to small changes in benefits during the study period. It is therefore possible that the changes in unemployment benefits during the study period were not sufficiently large to substantially affect CVD risk.

A further potential bias would arise if maximum unemployment benefits in a state changed in tandem with other secular changes. For example, unemployment benefits may have been extended in some states in response to increasing unemployment rates. If economic downturns are associated with higher CVD risk, this would underestimate the health benefits of unemployment compensation. To test this hypothesis, we incorporated in the models state-level unemployment rates for each year and experimented with a one-year lag with respect to the unemployment benefit levels (results not shown). The association between state-level unemployment benefits and cardiovascular disease risk remained unaffected, suggesting that this did not explain our results.

### Conclusion

Our study illustrates the potential of linking state-level policies to individual-level data to examine their impact on health. Our results suggest that changes in the generosity of maximum unemployment compensation benefits did not affect the risk of CVD in a sample of old American workers. Future studies should examine whether state unemployment benefits may influence other health outcomes more sensitive to financial strain in the short-term, or whether changes in policy at younger ages might be more important in shaping cardiovascular risk at older ages.
